# Parallel Detection of the Unamplified Carbapenem Resistance Genes *bla*_NDM-1_ and *bla*_OXA-1_ Using a Plasmonic Nano-Biosensor with a Field-Portable DNA Extraction Method

**DOI:** 10.3390/bios15020112

**Published:** 2025-02-14

**Authors:** Kaily Kao, Evangelyn C. Alocilja

**Affiliations:** 1 Department of Biosystems and Agricultural Engineering, Michigan State University, East Lansing, MI 48824, USA; kaokaily@msu.edu; 2 Global Alliance for Rapid Diagnostics (GARD), Michigan State University, East Lansing, MI 48824, USA

**Keywords:** diagnostics, biosensors, nanoparticles, carbapenem resistance genes, antimicrobial resistance, genotypic

## Abstract

Antimicrobial resistance (AMR) is a rapidly growing global concern resulting from the overuse of antibiotics in agricultural and clinical settings. The challenge is exacerbated by the lack of rapid surveillance for resistant bacteria in clinical, environmental, and food supply settings. The increasing resistance to carbapenems, an important sub-class of beta-lactam antibiotics, is a major concern in the healthcare community. Carbapenem resistance (CR) has been found in the environment and food supply chain, where it has the potential to spread to pathogens, animals, and humans through direct or indirect contact. Rapid detection for preventative and control measures should be developed. This study utilized a gold nanoparticle-based plasmonic biosensor for the parallel detection of the CR genes *bla*_NDM-1_ and *bla*_OXA-1_. To explore the field portability, DNA was extracted using two methods: a commercial extraction kit and a boiling method. The results were compared between the two methods using a spectrophotometer and a cellphone application for RGB values to quantify the visual results. The results showed that the boiling method of extraction was more effective than extraction with a commercial kit for this analysis. The parallel detection of unamplified genes extracted via the boiling method is novel. When combined with other portable testing equipment, the approach has the potential to be an inexpensive, rapid, and simple on-site CR gene detection protocol.

## 1. Introduction

Antimicrobial resistance (AMR) has emerged as one of the greatest global concerns of the 21st century, directly resulting in 1.14 million deaths and contributing to 4.71 million deaths in 2021 [1,2]. The overuse and improper use of antimicrobials contribute to the increase in antimicrobial resistance [3]. Antibiotics are critical in the healthcare field for the prevention and treatment of infections in patients, especially those with chronic diseases or who have had complex surgeries [4]. In addition to antibiotics used in healthcare, the agricultural industry is a large consumer of antibiotics for animal disease prevention and growth promotion [5]. The misuse of antibiotics in both industries has an impact on the increase in AMR [4,6]. Bacteria can develop several mechanisms of AMR, including the destruction of antibiotics using enzymes, changing the antibiotic target of inhibition, and pumping out antibiotics via efflux pumps [7]. In bacteria, resistance genes can be obtained through horizontal gene transfer from mobile genetic elements, like plasmids, or developed through spontaneous gene mutations [4]. Carbapenems are beta-lactam antibiotics that can treat infections involving most bacteria [8]. Among the beta-lactams, carbapenems are the most broad-spectrum antibiotics, with the presence of a beta-lactam ring along with a carbapenem [9]. This provides stability against beta-lactamases that inactivate beta-lactams. Carbapenems are considered last-resort antibiotics for serious bacterial infections [9]. Incidentally, carbapenem resistance has increased in the last few years [8]. Carbapenem resistance (CR) in Gram-negative bacteria has increased substantially in the last thirty years [2]. This increasing carbapenem resistance is a growing worldwide health concern [10].

The emergence of carbapenem-resistant bacteria (CRB) in the environment can stem from hospital environments and wastewater, even if properly treated [11]. CRB were shown to grow in wastewater treatment plants and survive the treatment process [11]. It was also shown that wildlife and companion animals could pass resistance genes to humans [11]. Although carbapenems are not used in agriculture or farm animal treatment, CRB do exist in animals and agricultural environments [12,13,14,15]. CRB containing the *bla*_KPC_ and *bla*_NDM_ genes were found in sewage and water samples in Brazil [12]. Multiple CRB were found in treated wastewater in European urban settings and Japanese environmental settings [13,15]. CRB were found in retail meat products and pig feces [14]. It is likely that the CRB originating in hospitals where carbapenems were used as treatments entered the environment through horizontal gene transfer and spread through water sources and animals [11]. The detection of CR genes in the environment and food supply chain is important as a preventative measure to avoid the spread of CR in humans [16].

The methods of CR detection include phenotypic and genotypic testing methods [17]. The goal of phenotypic testing is to observe the inhibition of bacterial growth in the presence of antibiotics, while genotypic testing aims to detect specific resistance genes [18]. The phenotypic tests include chromogenic-based media for screening purposes, the modified Hodge test, Carba NP, and traditional antibiotic susceptibility testing (AST) [17]. Traditional AST includes disc diffusion, broth dilution, and agar dilution [19,20,21,22,23]. The genotypic tests include PCR-based methods, hybridization-based techniques, and whole-genome sequencing (WGS) [10]. An alternative method of carbapenem-resistance detection is matrix-assisted laser desorption/ionization time-of-flight mass spectrometry (MALDI-TOF) [17]. MALDI-TOF has been used to detect carbapenem molecules and carbapenemase activity [24]. One common disadvantage of current phenotypic methods of detection of carbapenem resistance is the time required to complete the testing. Table 1 shows the estimated time required to complete each phenotypic and genotypic testing method.

Beta-lactams, and more specifically, carbapenemase enzymes, are broken down into classes according to the primary genetic sequences, including Class A, Class B, and Class D [10,36]. Class A includes classical narrow-spectrum, extended-spectrum beta-lactamase (TEM, SHV, CTX-M) and Class A carbapenemases (KPC, GES, IMI) [36]. Of these, KPCs are the most common [10]. Class B carbapenemases are susceptible to ethylenediaminetetraacetic acid (EDTA) and includes metallo-beta-lactamase (IMP, VIM, NDM) [36]. The most common among these is New Delhi metallo-beta-lactamase 1 (*bla*_NDM-1_) [10]. Class D carbapenemases are poorly inhibited by EDTA and include extended-spectrum beta-lactamase (OXA) [36]. One example of a Class D carbapenemase is *bla*_OXA-1_ [37].

Gold nanoparticles have gained attention for use in plasmonic biosensors due to their multifunctional characteristics in terms of diagnostics, therapeutics, and imaging [38]. GNPs possess surface plasmon resonance (SPR) properties due to the oscillation of free electrons influenced by their size and shape, and this property can be utilized in colorimetric biosensing assays [39]. This optical biosensor uses dextrin-capped GNPs coated with 11-mercaptoundecanoic acid (MUDA, HSCH_2_(CH_2_)_8_CH_2_OOH), where the thiol end of the MUDA attaches to the GNP surface and its carboxyl group binds to the amine component of an aminated oligonucleotide probe. This probe binds to the specific target sequence when target DNA is present, encapsulating the GNPs within the genomic structure. Upon the addition of hydrochloric acid (HCl), the GNPs are protected from agglomeration. Small, dispersed GNPs have a maximum oscillation range around 520 nm and appear red in color [40]. When GNPs aggregate, the maximum oscillation range shifts to the right, causing a visible color change from red to blue. This optical biosensor can produce colorimetric results, distinguishing a target sample from a non-target sample based on the level of aggregation of the GNPs. This method does not require gene amplification, and the assay can be completed within 45 min after DNA extraction. This study aimed to develop a rapid assay for the parallel detection of several CR genes in bacteria, utilizing a plasmonic DNA-based biosensor. The novelty lies in the parallel detection using a simple boiling method for a potential field-operable system.

## 2. Materials and Methods

### 2.1. Materials

Frozen susceptible bacterial stock cultures of *Klebsiella pneumoniae* (*K. pneumoniae*), *Enterobacter cloacae* (*E. cloacae*), *Staphylococcus aureus* (*S. aureus*), and *Escherichia coli* (*E. coli*) C-3000 were obtained from the Nano-Biosensors Laboratory at Michigan State University. Carbapenem-resistant strains of *E. coli* BAA-2471 and *E. coli* BAA-2340 were obtained from the American Type Culture Collection (ATCC). The DNeasy Powerlyzer Microbial Kit (Qiagen, Germantown, MD, USA) was used for the DNA extraction. A NanoDrop One spectrophotometer (Thermo Fisher Scientific, Waltham, MA, USA) was used to measure the absorbance spectra and quantify the DNA concentration extracted using the kit. A Qubit Fluorometric Quantification device (Thermo Fisher Scientific) was used to quantify the DNA concentration extracted via boiling. Oligonucleotide probes were ordered from Integrated DNA Technologies (IDT). Tryptic Soy Agar (TSA) and Tryptic Soy Broth (TSB), hydrochloric acid, gold (III) chloride, sodium carbonate, 11-mercaptoundecanoic acid, sodium dodecyl sulfate, and dextrin from potato starch were purchased from Sigma Aldrich (St. Louis, MO, USA).

### 2.2. Bacterial Cultures

The frozen stock cultures of bacteria were stored at −80 °C. Bacteria from the stock cultures were streaked on the TSA and incubated at 37 °C for 24 h to create master plates. These were stored at 4 °C for a maximum of 4 weeks before replacement. Bacterial broth cultures were created for each experiment by transferring one colony into 9 mL of TSB and allowing it to grow overnight.

### 2.3. Oligonucleotide Probe Design and PCR Verification

The single-stranded oligonucleotide probes were designed to target specific CR genes, *bla*_NDM-1_ and *bla*_OXA-1_. The probe sequences were designed using tools from the National Center for Biotechnology Information Basic Location Alignment Search Tool (NCBI BLAST). NCBI BLAST confirmed that the probe sequence was specific to organisms containing the respective target gene. The complementary target strand binds to the specific bases of the probe, which means adenine (A) binds to thymine (T) and guanine (G) binds to cytosine (C) across the sequence. The probe sequences, lengths, and melting temperatures (Tm) are shown in Table 2.

The presence or absence of resistance genes in the bacterial pure cultures was confirmed with PCR, followed by gel electrophoresis. Each resistance gene utilized different forward and reverse primers, and separate methods were developed based on previous studies. The forward and reverse primers and DNA probe sequences were confirmed to be located within the gene of interest. Note that PCR analysis was used as a verification method for the selectivity of the DNA probe. The biosensor assay utilized unamplified DNA.

The PCR detection of the *bla*_NDM-1_ gene used a method from a previous study [41]. The PCR reaction mixture included 12.5 μL of PCR Master Mix reagent (Qiagen), 0.5 μL of 10 μM F-primer, 0.5 μL of 10 μM R-primer, 10.5 μL of water, and 1 μL of DNA template for a total reaction volume of 25 μL [41]. The thermal cycling conditions included an initial heating step at 94 °C for 2 min, followed by 35 cycles of 94 °C for 30 s, 55 °C for 30 s, and 72 °C for 45 s, and a final extension step at 72 °C for 10 min [41]. The PCR products were separated on 1.8% agarose gel after staining with 6x loading dye and Sybr. The forward and reverse primer sequence, length, and melting temperature are listed in Table 2.

The PCR detection of the *bla*_OXA-1_ gene used forward and reverse primers from a previous study [42]. The PCR reaction mixture included 12.5 μL of PCR Master Mix reagent (Qiagen), 2 μL of 2 μM F-primer, 2 μL of 2 μM R-primer, 7.5 μL of water, and 1 μL of DNA template for a total reaction volume of 25 μL. The thermal cycling conditions included an initial heating step at 94 °C for 5 min, followed by 32 cycles of 94 °C for 30 s, 52 °C for 30 s, and 72 °C for 60 s, and a final extension step at 72 °C for 10 min [42]. The PCR products were separated on 2.0% agarose gel after staining with 6× loading dye and Sybr. The forward and reverse primer sequence, length, and melting temperature are listed in Table 2.

### 2.4. GNP Synthesis and Modification

**Figure 1:**
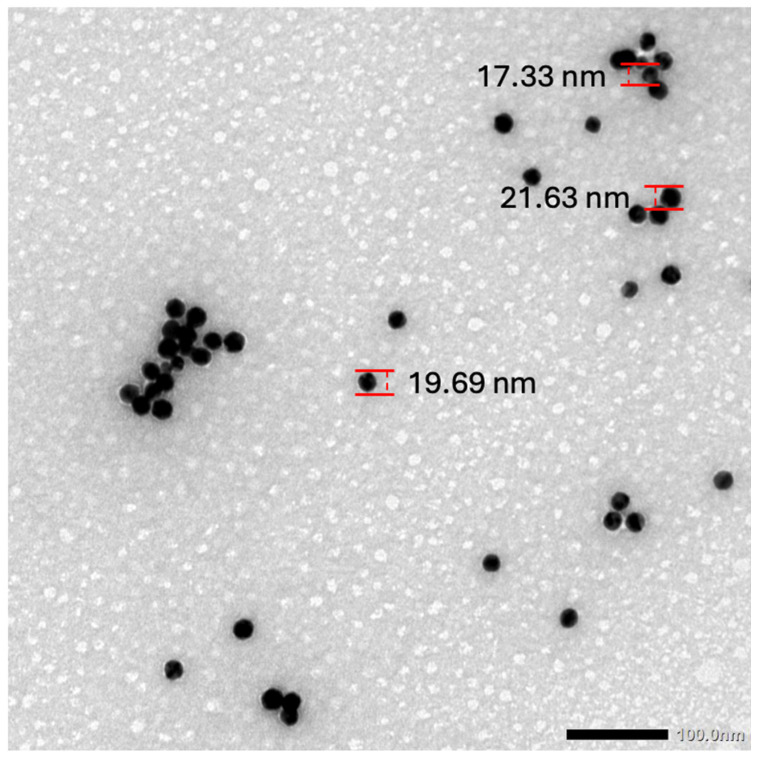
TEM image of GNPs used for the diameter measurement at the 100 nm scale.

Dextrin-capped gold nanoparticles (GNPs) were synthesized according to a method previously described [43]. The materials used were gold (III) chloride trihydrate (HAuCl4), sodium carbonate (Na_2_CO_3_), dextrin, and sterile water. The quality of the GNPs was confirmed by measuring the maximum absorbance from 518–522 nm using the NanoDrop One (version 2.9). To characterize the size of the GNPs, TEM images were taken and a sample of 40 GNPs were measured, using ImageJ software version 1.54 g [44]. The average diameter was 19.64 ± 1.39 nm and ranged from 16.61 to 22.53 nm. Figure 1 shows the TEM image where the measurements were taken.

**Figure 2:**
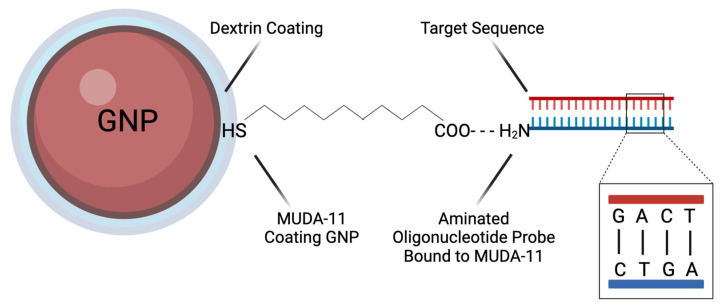
A dextrin-capped GNP is coated with MUDA-11, which is bound to an aminated oligonucleotide probe. If the target sequence is present in the sample, it binds with the probe.

The GNPs were thiol-coated using 11-mercaptoundecanoic acid (MUDA) and then suspended in borate buffer. The GNP-MUDA was standardized to have a final absorbance of 520 nm by controlling the borate buffer volume. The GNPs were stored at 4 °C before use. Figure 2 shows the dextrin-capped, thiol-coated GNP interaction with an aminated oligonucleotide probe and target sequence of DNA. Note that the target sequence that binds to the probe is only a portion of an extracted bacterial genome.

### 2.5. Biosensor Design, Principle, and Optimization

**Figure 3:**
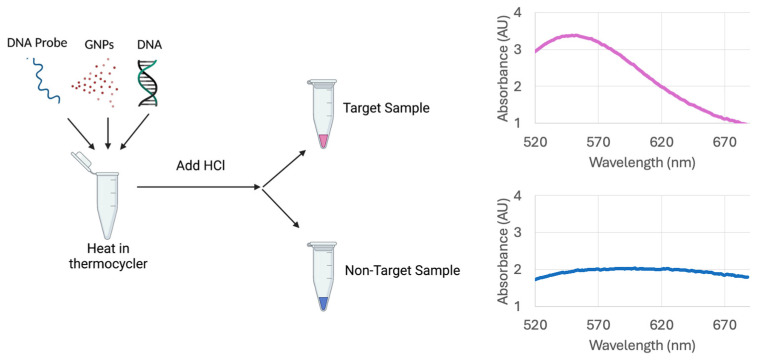
GNP-based biosensor detection of target DNA sequences utilizing an oligonucleotide probe. Visual results can be quantified using wavelength and absorbance measurements taken with a spectrophotometer. The target samples have an absorbance peak near 520 nm, corresponding to a red sample. The non-target samples result in a shift of the peak absorbance near 620 nm, which corresponds to a blue sample.

The plasmonic biosensor utilizes GNPs, single-stranded oligonucleotide probes, and sample DNA. To each sample tube, 5 μL of probe, 5 μL of GNPs, and 10 μL of sample DNA were combined and pipette mixed. The oligonucleotide probes were diluted to a concentration of 25 μM. The sample DNA was diluted to either 20 ng/μL or 2.5 ng/μL depending on the extraction method detailed below. The sample tubes were placed in a thermocycler to denature the sample DNA for 5 min at 95 °C, anneal the DNA to the probe for 10 min at 55 °C, and cool for 1 min at 27 °C. Then, the optimized volume of HCl was added. This general principle and the methods are shown in Figure 3.

The biosensor is based on the unique surface plasmon resonance (SPR) properties of GNPs, which are caused by the coherent oscillation of free electrons [45]. This oscillation reaches a maximum at a specific frequency or SPR, and this induces a strong absorption of light that can be measured using a spectrophotometer [45]. The SPR band intensity and maximum depend on the size and shape of the GNPs [45,46]. Other studies have shown that GNPs smaller than 30 nm have a maximum oscillation range of 500 nm to 540 nm [46,47]. The GNPs used in this assay are 16.61–22.53 nm in diameter, with an absorption wavelength from 518 to 522 nm. When GNPs aggregate, it causes a shift in the SPR band and results in a visible color change [46]. This property is used in this assay to distinguish between a target (positive) and a non-target (negative) sample after adding HCl to induce agglomeration. The MUDA-coated, dextrin-capped GNPs link with the aminated probe to create a GNP–probe complex. It is hypothesized that when the target DNA sequences bind to the probe, the target–probe binding will prevent the GNPs from aggregating, which indicates a positive sample. When the GNPs aggregate, it indicates a negative sample. The distance-dependent nature of the SPR band allows this color change to be measured using a spectrophotometer.

The volume of HCl added was studied and optimized. The optimal HCl volume was determined by adding different amounts of HCl, starting at 4 μL and increasing to 8 μL in 1 μL increments to separate the trials containing target, non-target, and control samples. The optimal volume occurred when the target sample remained visibly red, and the non-target sample and control were blue after 10 min. Following optimization, spectral measurements for testing were taken at 10 min. To quantitatively analyze the spectral data, the absorbance values at 520 nm and 620 nm were compared in a ratio (A_520_/A_620_) to describe how red or blue each sample was. At 520 nm, red light is reflected and green light is absorbed. At 620 nm, orange light is absorbed and blue light is reflected.

### 2.6. DNA Extraction Methods

This study compared two DNA extraction methods. The purpose was to evaluate the efficiency of a commercial DNA extraction kit versus a boiling method. DNA was extracted using the DNeasy Powerlyzer Microbial Kit (Qiagen), and the extraction method followed the manufacturer’s protocol. The DNA purity and concentration extracted using the kit were measured using the Nanodrop One (Thermo Fisher Scientific, Waltham, MA, USA). For the boiling method, an overnight pure culture of bacteria was centrifuged at 8000 rpm for 3 min. Then, the supernatant was removed, and the cell pellet was resuspended in elution buffer 1x AE. Next, the tubes were boiled at 99 °C for 20 min. Immediately after boiling, the tubes were placed inside the freezer at −20 °C for 5 min. After centrifuging at 15,000 rpm for 2 min, the top half of the supernatant was collected and used for biosensing applications. The DNA purity was quantified using the Nanodrop One. The quantity of DNA was measured using a Qubit Fluorometric Quantification device (Thermo Fisher Scientific, Waltham, MA, USA).

### 2.7. Selectivity and Limit of Detection Testing

A series of 5 trials for each probe, using DNA extracted with a commercial kit and DNA extracted through boiling, was conducted to determine the biosensor’s selectivity. For the DNA extracted with a kit, the DNA concentrations were standardized to 20 ng/μL using a Nanodrop One. The concentration of DNA extracted through boiling was standardized to 2.5 ng/μL using a Qubit Fluorometer (Thermo Fisher Scientific, Waltham, MA, USA) to achieve more accurate double-stranded DNA concentrations due to the non-purified DNA extracts used. The target species used for the *bla*_NDM-1_and *bla*_OXA-1_ probes was *E. coli* BAA-2471. Four non-target bacterial species were used for all the oligonucleotide probe analyses: *E. coli* C-3000, *S. aureus*, *E. cloacae*, and *K. pneumoniae*. A negative control of nuclease-free water was also included. DNA extraction was performed using the Qiagen Powerlyzer Microbial kit on overnight pure bacterial transfers. The extracted DNA concentration was measured using the Nanodrop One dsDNA program and diluted to 20 ng/μL. DNA was also extracted using a boiling extraction procedure, and the DNA was further diluted to 2.5 ng/μL using a Qubit Fluorometric Quantification device. An optimized amount of HCl was added to each sample, and after ten minutes, the absorbance was measured with the Nanodrop One. The results were analyzed through a ratio of the absorbance at 520 nm and 620 nm. Target samples A_520_/A_620_ were compared to non-target samples A_520_/A_620_ to determine whether they were significantly different from each other (alpha = 0.05). In addition to the spectral measurements, pictures of each test run were taken at the optimal time and analyzed using a cellphone application. The cellphone application measures the images’ red/green/blue (RGB) values, giving an alternative quantitative analysis of how red or blue a sample is. A series of 5 trials for each probe was conducted to determine the biosensor’s limit of detection (LOD). All the bacterial DNA was extracted using the same method as mentioned above. The DNA was diluted to lower concentrations ranging from 20 to 1.25 ng/μL for samples extracted using the commercial kit and 2.5 to 0.15625 ng/μL for samples extracted through boiling. For each trial, the sample containing the target DNA was compared to a non-target sample (*E. coli* C-3000), of the same DNA concentration, and a control (nuclease-free water). The optimized amount of HCl was added, and after ten minutes, the absorbance was measured with the Nanodrop One. The A_520_/A_620_ for the target, non-target, and control samples were compared to determine when the target sample was no longer distinguishable from the non-target at the same concentration or the control.

### 2.8. Statistical Analysis

The statistical analyses utilized the 95% confidence intervals of the A_520_/A_620_ ratio to compare the target and non-target sample results concerning selectivity. The LOD testing used the 95% confidence intervals of the A_520_/A_620_ ratio to compare the target and non-target samples at different DNA concentrations. All the tests of the selectivity and LOD had a sample size of 5 replicates (*n* = 5), and the confidence intervals were calculated using Student’s t distribution and the standard error.

## 3. Results

### 3.1. Optimization of Biosensor DNA Probes with DNA Extracted with and Without a Commercial Extraction Kit

**Figure 4:**
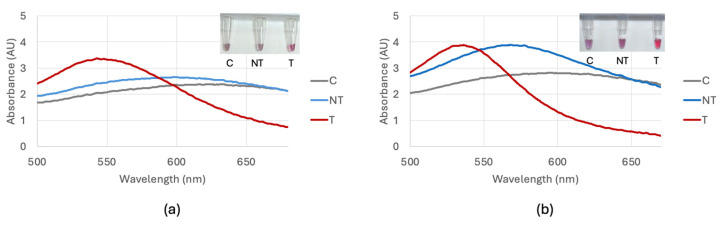
(**a**) Optimization of the bla_NDM-1_ probe 1 visual and spectral result for 6 μL comparing the control (C), non-target *E. coli* C-3000 (NT), and target *E. coli* BAA-2471 (T) using DNA extracted with a DNA extraction kit. (**b**) Optimization of the bla_NDM-1_ probe 1 visual and spectral result for 5 μL comparing the control (C), non-target *E. coli* C-3000 (NT), and target *E. coli* BAA-2471 (T) using DNA extracted through boiling.

**Figure 5:**
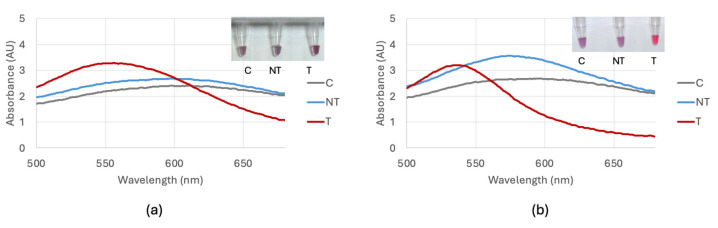
(**a**) Optimization of the bla_NDM-1_ probe 2 visual and spectral result for 7 μL comparing the control (C), non-target *E. coli* C-3000 (NT), and target *E. coli* BAA-2471 (T) using DNA extracted with a DNA extraction kit. (**b**) Optimization of the bla_NDM-1_ probe 2 visual and spectral result for 6 μL comparing the control (C), non-target *E. coli* C-3000 (NT), and target *E. coli* BAA-2471 (T) using DNA extracted through boiling.

**Figure 6:**
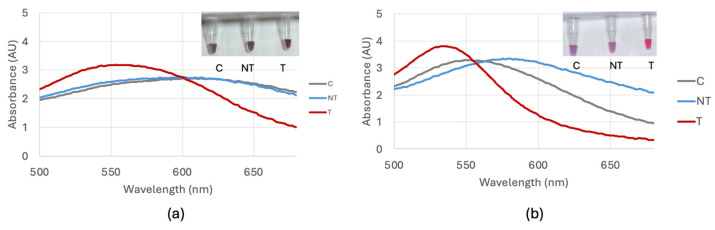
(**a**) Optimization of the bla_OXA-1_ probe visual and spectral result for 7 μL comparing the control (C), non-target *E. coli* C-3000 (NT), and target *E. coli* BAA-2471 (T) using DNA extracted with a DNA extraction kit. (**b**) Optimization of the bla_OXA-1_ probe visual and spectral result for 6 μL comparing the control (C), non-target *E. coli* C-3000 (NT), and target *E. coli* BAA-2471 (T) using DNA extracted through boiling.

Optimization of the biosensor using DNA extracted with a kit resulted in the addition of 6 μL of 0.1 M HCl for the bla_NDM-1_ probe 1 and 7 μL of 0.1 M HCl for the bla_NDM-1_ probe 2 and bla_OXA-1_ probe. Optimization of the biosensor using DNA extracted through the boiling method resulted in the addition of 5 μL for the bla_NDM-1_ probe 1 and 6 μL for the bla_NDM-1_ probe 2 and bla_OXA-1_ probe. The time between the HCl addition and a visible color change was 10 min. Figure 4 shows a comparison of the optimization of the bla_NDM-1_ probe 1 comparing target *E. coli* BAA-2471, non-target *E. coli* C-3000 and control nuclease-free water with DNA extracted with and without a kit. Optimization of the bla_NDM-1_ probe 2 utilized the same methods as the bla_NDM-1_ probe 1 and is shown in Figure 5. Optimization of the bla_OXA-1_ probe is shown in Figure 6.

### 3.2. Selectivity Testing of Pure Bacterial Culture DNA Extracted with and Without a Commercial Extraction Kit

**Figure 7:**
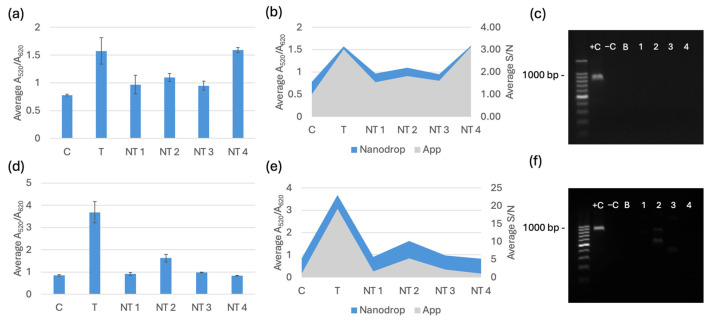
(**a**) Average A_520_/A_620_ for the *bla*_NDM-1_ probe 1 with DNA from a kit 10 min after 0.1 M HCl addition. (**b**) App data average S/N values compared to the A_520_/A_620_ for the *bla*_NDM-1_ probe 1 with DNA extracted with a kit 10 min after 0.1 M HCl addition. (**c**) PCR verification for *bla*_NDM-1_ using DNA extracted with a kit. (**d**) Average A_520_/A_620_ data of the selectivity analysis for the *bla*_NDM-1_ probe 1 with DNA extracted through boiling 10 min after 0.1 M HCl addition. (**e**) App data average S/N values compared to the A_520_/A_620_ for the *bla*_NDM-1_ probe 1 with DNA extracted through boiling 10 min after 0.1 M HCl addition. (**f**) PCR verification for *bla*_NDM-1_ using DNA extracted via boiling.

**Figure 8:**
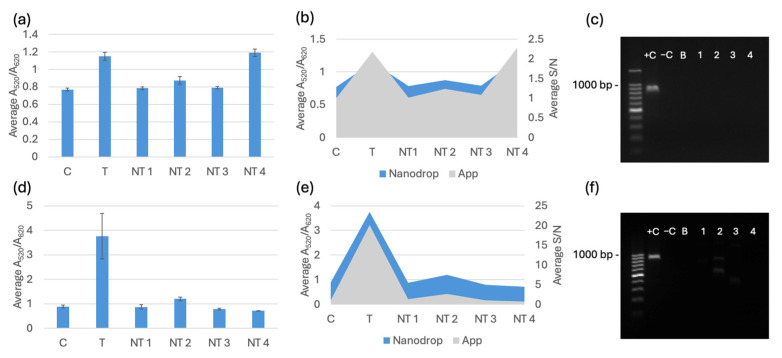
(**a**) Average A_520_/A_620_ for the *bla*_NDM-1_ probe 2 with DNA from a kit 10 min after 0.1 M HCl addition. (**b**) App data average S/N values compared to the A_520_/A_620_ for *bla*_NDM-1_ probe 2 with DNA extracted with a kit 10 min after 0.1 M HCl addition. (**c**) PCR verification for *bla*_NDM-1_ using DNA extracted with a kit. (**d**) Average A_520_/A_620_ data of the selectivity analysis for the *bla*_NDM-1_ probe 2 with DNA extracted through boiling 10 min after 0.1 M HCl addition. (**e**) App data average S/N values compared to the A_520_/A_620_ of the selectivity analysis for the *bla*_NDM-1_ probe 2 with DNA extracted through boiling10 min after 0.1 M HCl addition. (**f**) PCR verification for *bla*_NDM-1_ with DNA extracted via boiling.

**Figure 9:**
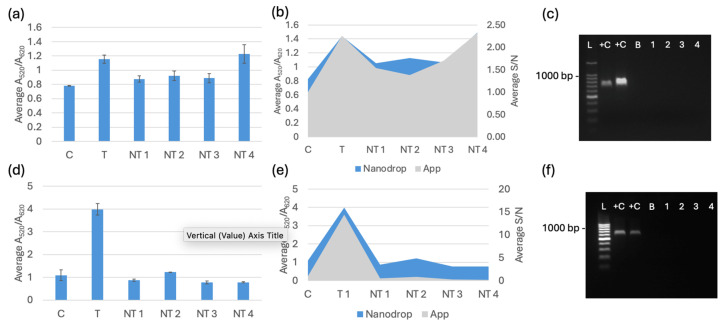
(**a**) Average A_520_/A_620_ for the *bla*_OXA-1_ probe with DNA from a kit 10 min after 0.1 M HCl addition. (**b**) App data average S/N values compared to the A_520_/A_620_ for the *bla*_OXA-1_ probe with DNA extracted with a kit 10 min after 0.1 M HCl addition. (**c**) PCR verification for *bla*_OXA-1_ using DNA extracted with a kit. (**d**) Average A_520_/A_620_ data of the selectivity analysis for the *bla*_OXA-1_ probe with DNA extracted through boiling 10 min after 0.1 M HCl addition. (**e**) App data average S/N values compared to the A_520_/A_620_ of the selectivity analysis for *bla*_OXA-1_ probe with DNA extracted through boiling 10 min after 0.1 M HCl addition. (**f**) PCR verification for *bla*_OXA-1_ with DNA extracted via boiling.

The selectivity testing for the biosensor was completed after optimization. Each trial included a negative control of nuclease-free water, a target sample, and four non-target species: *E. coli* C-3000 (NT1), *K. pneumoniae* (NT2), *E. cloacae* (NT3), and *S. aureus* (NT4). The target for *bla*_NDM-1_ and *bla*_OXA-1_ was *E. coli* BAA-2471. All the DNA extracted from the kit was diluted to 20 ng/μL before testing. All the DNA extracted from boiling was diluted to 2.5 ng/μL before testing. The spectral results are displayed alongside the RGB phone application measurements and PCR verification in Figure 7, Figure 8 and Figure 9. Figure 7a, Figure 8a and Figure 9a show the average spectral A_520_/A_620_ with DNA extracted with a kit comparing the control (C) nuclease-free water, non-targets *E. coli* C-3000 (NT1), *K. pneumoniae* (NT2), *E. cloacae* (NT3), and *S. aureus* (NT4) and target *E. coli* BAA-2471 (T) 10 min after 0.1 M HCl addition. Figure 7b, Figure 8b and Figure 9b show the app data average S/N values compared to the A_520_/A_620_ of the selectivity analysis with DNA extracted with a kit comparing the control (C) nuclease-free water, non-targets *E. coli* C-3000 (NT1), *K. pneumoniae* (NT2), *E. cloacae* (NT3), and *S. aureus* (NT4) and target *E. coli* BAA-2471 (T) 10 min after 0.1 M HCl addition. Figure 7c and Figure 8c show the PCR verification, including a standard 100 bp ladder (Promega, Madison, WI, USA), positive control *E. coli* BAA-2471 (+C), negative control *E. coli* BAA-2340 (−C), blank AE buffer (B), non-target *S. aureus* (1), non-target *K. pneumoniae* (2), non-target *E. coli* C-3000 (3), and non-target *E. cloacae* (4) extracted from a kit. Figure 7d, Figure 8d and Figure 9d show the A_520_/A_620_ data of the selectivity analysis with DNA extracted through boiling comparing the control (C) nuclease-free water, non-targets *E. coli* C-3000 (NT1), *K. pneumoniae* (NT2), *E. cloacae* (NT3), and *S. aureus* (NT4) and target *E. coli* BAA-2471 (T) 10 min after 0.1 M HCl addition. Figure 7e, Figure 8e and Figure 9e show the app data average S/N values compared to the A_520_/A_620_ of the selectivity analysis for DNA extracted with a kit comparing the control (C) nuclease-free water, non-targets *E. coli* C-3000 (NT1), *K. pneumoniae* (NT2), *E. cloacae* (NT3), and *S. aureus* (NT4) and target *E. coli* BAA-2471 (T) 10 min after 0.1 M HCl addition. Figure 7f and Figure 8f show the PCR verification, including a standard 100 bp ladder (Promega, Madison, WI, USA), positive control *E. coli* BAA-2471 (+C), negative control *E. coli* BAA-2340 (–C), blank AE buffer (B), non-target *S. aureus* (1), non-target *K. pneumoniae* (2), non-target *E. coli* C-3000 (3), and non-target *E. cloacae* (4) extracted from boiling. Figure 9 follows the same pattern as Figure 7 and Figure 8; however, the PCR results in Figure 9c,f show the PCR verification, including a standard 100 bp ladder (Promega), positive control *E. coli* BAA-2471 (+C), positive control *E. coli* BAA-2340 (+C), blank AE buffer (B), non-target *S. aureus* (1), non-target *K. pneumoniae* (2), non-target *E. coli* C-3000 (3), and non-target *E. cloacae* (4) extracted from boiling.

The carbapenem-resistant bacterial pure cultures extracted with the commercial DNA extraction kit and through boiling were successfully detected by the *bla*_NDM-1_ probe 1, *bla*_NDM-1_ probe 2, and *bla*_OXA-1_ probe according to the statistical analysis and comparison of the 95% confidence intervals. *E. coli* C-3000 (NT1), *K. pneumoniae* (NT2), and *E. cloacae* (NT3) were successfully differentiated, using the average A_520_/A_620_ and application-measured RGB values, from the target sample for all the gene probes when using DNA extracted with the kit. *S. aureus* (NT4) was not differentiable from the target when DNA was extracted with a kit. For DNA extracted using boiling extraction, all the non-targets were successfully differentiated, using the average A_520_/A_620_ and application-measured RGB values, from the target samples of all three probes. The PCR gel electrophoresis for both extraction methods confirmed that the respective targets contained the target genes and the non-targets did not contain target genes. The false positive in the DNA-extracted kit was not observed with boiling extraction, and there were no other false positives. The average RGB application values were compared to the A_520_/A_620_ values from the spectrophotometer. A similar data trend was observed between the two measurement methods. The app has the potential to be used in field testing to avoid having to use a Nanodrop One or another spectrophotometer.

### 3.3. Limit of Detection Testing

**Figure 10:**
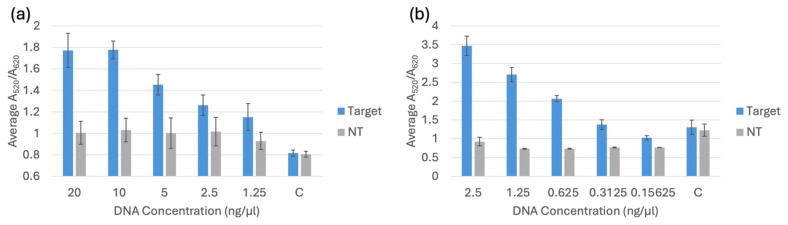
(**a**) Average A_520_/A_620_ for the *bla*_NDM-1_ probe 1 comparing different concentrations of target and non-target (NT) DNA extracted using a commercial kit and a negative control (C). (**b**) Average A_520_/A_620_ for the *bla*_NDM-1_ probe 1 comparing target and non-target (NT) DNA extracted through boiling and a negative control (C).

**Figure 11:**
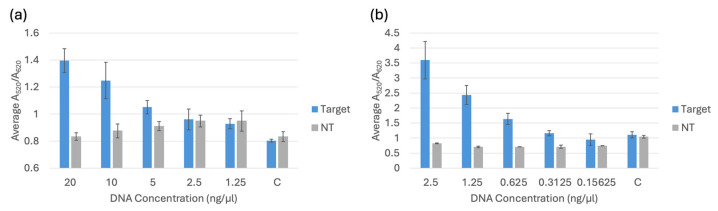
(**a**) Average A_520_/A_620_ for the *bla*_NDM-1_ probe 2 comparing different concentrations of target and non-target (NT) DNA extracted using a commercial kit and a negative control (C). (**b**) Average A_520_/A_620_ for the *bla*_NDM-1_ probe 2 comparing target and non-target (NT) DNA extracted through boiling and a negative control (C).

**Figure 12:**
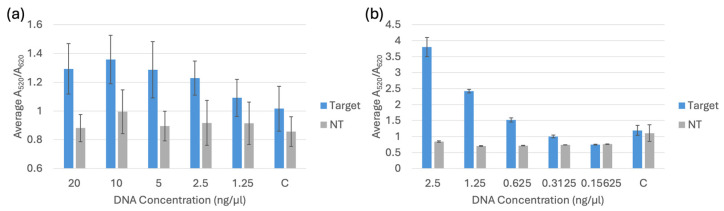
(**a**) Average A_520_/A_620_ for the *bla*_OXA-1_ probe comparing different concentrations of target and non-target (NT) DNA extracted using a commercial kit and a negative control (C). (**b**) Average A_520_/A_620_ for the *bla*_OXA-1_ probe comparing target and non-target (NT) DNA extracted through boiling and a negative control (C).

Limit of detection testing for the biosensor was used to determine the lowest detectable concentration of target DNA when compared to non-target DNA of the same concentration. Each trial included a negative control of nuclease-free water, a set of target DNA samples, and a set of non-target DNA samples. The target and non-target DNA were diluted from 20 ng/μL to 1.25 ng/μL for DNA extracted using a commercial kit and from 2.5 ng/μL to 0.15625 ng/μL for DNA extracted through boiling. The target for *bla*_NDM-1_ and *bla*_OXA-1_ was *E. coli* BAA-2471. For all the trials, *E. coli* C-3000 was used as the non-target for comparison. The spectral results of the A_520_/A_620_ ratios are displayed in Figure 10, Figure 11 and Figure 12. The LOD of each probe was determined by analyzing the overlap of the 95% confidence intervals. The LOD occurred when the target sample was no longer distinguishable from the non-target or the control sample, whichever caused the higher limit of detection.

By comparing the 95% confidence intervals of the target, non-target and control samples, it was statistically determined that the LOD for the *bla*_NDM-1_ probe 1 was 1.25 ng/μL for DNA extracted with the kit and 0.625 ng/μL for DNA extracted through boiling. The LOD for the *bla*_NDM-1_ probe 2 was 5 ng/μL for DNA extracted with the kit and 0.625 ng/μL for DNA extracted through boiling. The LOD for the *bla*_OXA-1_ probe was 2.5 ng/μL for DNA extracted with the kit and 0.625 ng/μL for DNA extracted through boiling. For the sample DNA extracted with the kit, the target samples were compared to the non-target samples of the same concentration to determine the LODs, and for the sample DNA extracted through boiling, the target samples were compared to the controls. The controls for the boiling method had a higher response than the non-targets at all the concentrations. Therefore, they were used as a comparison to avoid a lower LOD that could potentially cause interference between a target and a blank.

## 4. Discussion

The rapid detection of antimicrobial resistance genes is necessary in environments such as farms and food processing plants for the prevention of the spread of carbapenem resistance [16]. Due to their clinical importance, resistance to carbapenem antibiotics is especially concerning [48]. The current “gold standard” phenotypic methods of carbapenem-resistance detection, including broth dilution, agar dilution, and disc diffusion, are time-consuming and require cultivable organisms [27,28,29,30,31]. PCR-based methods are rapid; however, they are relatively expensive, have complicated procedures, and are sensitive to external conditions [10,32]. Biosensing technology shows promise for the development of rapid, inexpensive, and accurate detection methods for multiple antibiotic-resistance genes [49].

Using this DNA-based biosensor, two CR genes, *bla*_NDM-1_ and *bla*_OXA-1_, were detected specifically compared to four susceptible bacterial non-targets. These non-targets included *E. coli* C-3000, *K. pneumoniae*, *E. cloacae*, and *S. aureus*. These non-targets were selected to include some of the Enterobacterales ESKAPE group of bacterial species. This group causes a high percentage of resistant human infections and includes *Enterococcus faecium*, *Staphylococcus aureus*, *Klebsiella pneumoniae*, *Acinetobacter baumannii*, *Pseudomonas aeruginosa*, and *Enterobacter cloacae* [50]. Susceptible ESKAPE pathogens were included as non-targets to ensure the biosensor is specific to the gene even in the presence of commonly carbapenem-resistant organisms. Boiling DNA extraction resulted in a more specific outcome. *S. aureus* did not contain either *bla*_NDM-1_ or *bla*_OXA-1_ according to the PCR results shown, and it did not cause a false positive response in the biosensor when using DNA extracted through boiling. To verify that the PCR results were consistent with the biosensor results, all the probe sequences and primers were confirmed to be within the gene of interest. The respective forward and reverse primers were located in the complete gene sequence provided by the American Type Culture Collection (ATCC) for each target gene. Once the primers and probe sequences were located within the gene, the exact amplicon length could be calculated, and it was confirmed that the detected amplified sequence in the PCR matches with the target sequence of the probe detected with the biosensor. Therefore, the presence or absence of the gene sequence is consistent between the PCR analysis and the biosensor assays. The reported amplicon lengths for the *bla*_NDM-1_ primers were 100–200 bp [41]. However, when comparing the primer separation in the full gene sequence provided by the ATCC, the actual amplicon length was 798 bp. This is consistent with the PCR gel electrophoresis results for *bla*_NDM-1_. The reported amplicon length for *bla*_OXA-1_ was 619 bp [42]. This was confirmed by the separation of primers in the known sequence and with the PCR gel electrophoresis results. Since boiling extraction was proven to result in a more specific analysis, it was concluded that it was the better DNA extraction option for this assay.

The LOD for this assay, depending on the DNA extraction method, ranged from 0.625 ng/μL to 5 ng/μL for the *bla*_NDM-1_ probe 1, *bla*_NDM-1_ probe 2, and *bla*_OXA-1_ probe. This is less than or near the detection limits of similar assays. One study detecting *Pseudomonas syringae* using thiol-linked DNA-GNPs had a detection limit of 15 ng/μL [51]. Another study detected the thermonuclease gene in *S. aureus* with a detection limit of 2.5 ng/μL [52]. A similar study detected the *inv*A gene in *Salmonella* with an LOD of 21.78 ng/μL [53]. Another study detected pathogenic *Mycobacteria* with an LOD of 18.75 ng/μL [54].

One potential roadblock in terms of all the DNA-based biosensor assays is DNA extraction. DNA extraction involves lysing bacterial cells and isolating their genomic material without contaminants like RNA and protein [55]. Powerlyzer DNA Extraction kits were utilized successfully in other studies for isolating DNA [52,56,57]. DNA extraction through boiling was utilized successfully in other studies as well [58,59,60]. The boiling method reduced the overall contamination and processing time [58]. The boiling method was compared with several commercial extraction kits and was found to be more rapid, less expensive, and not require special reagents [61]. Depending on the commercially available DNA extraction kits used, there can be different percentages of chromosomal and plasmid DNA recovery [62]. More than 80% of carbapenem resistance genes are located on plasmids and can be easily transferred to other organisms [63]. Both resistance genes *bla*_NDM-1_ and *bla*_OXA-1_ are found on plasmids [64,65]. It has been shown that plasmids and chromosomes can participate in gene transfer with each other, but in one study, only about 5% of the AMR genes were shared between the plasmids and their host chromosome [66]. Another study found that some cells that acquired plasmids with CR genes then integrated the plasmid with the chromosome until exposed to a carbapenem, which resulted in the release of the plasmids [67]. Another study found that 33% of the AMR genes were shared between the plasmids and the chromosomes of *Escherichia*, *Salmonella*, and *Klebsiella*, which could indicate the lateral gene transfer of AMR genes [68]. Some commercial kits are not designed for the extraction of plasmid DNA, which could result in false negative results and potentially cause issues in future testing. The location of the target gene and the DNA extraction method could potentially influence the amount of the target DNA successfully extracted from bacterial cells, which could impact the biosensing capabilities. This has not yet been thoroughly studied, and this biosensing technology could potentially benefit from this information.

## 5. Conclusions

The GNP-based plasmonic biosensor assay detected the CR genes *bla*_NDM-1_ and *bla*_OXA-1_. To improve the portability and accessibility of the biosensor assay, the methods were adjusted for DNA extraction that did not require a kit, a portable thermal cycler machine, and an RGB measurement cellphone application. A boiling extraction method was utilized to isolate bacterial DNA and shown to be more effective in differentiating target and non-target bacteria. The boiling extraction method was simple and less time-consuming than using a kit for DNA extraction. The biosensor provided visual differentiation between target bacteria containing resistance genes and non-target bacteria within 45 min after DNA extraction. To quantitatively analyze the results, spectral data were analyzed using the ratio of absorbance values at 520 nm and 620 nm (A_520_/A_620_), in addition to a cellphone application measuring the RGB values.

For future work, individual probes will be combined in a multi-array experimental design to test several CR genes from one sample. Testing will also be performed to detect the *bla*_KPC_ CR gene. This will decrease the total assay time required to assess the type of carbapenem resistance a sample may contain. This proof-of-concept biosensor assay will be validated using carbapenem-resistant clinical isolates and DNA extracted from sample matrices.

## Figures and Tables

**Table 1:** Overview of the current phenotypic and genotypic methods of CR detection and the time required, advantages, and disadvantages of each method.

Detection Method	Time Required	Advantages	Disadvantages	Sources
Modified Hodge test	24–48 h	High sensitivity, simple procedure	Lack of specificity, time-consuming	[25]
Carba NP	2 h	Rapid, high specificity	Inconsistent sensitivities	[26]
Traditional AST	24–48 h	Inexpensive, quantitative or qualitative	Laborious, error-prone methods, time-consuming	[27,28,29,30,31]
PCR-based methods	4–6 h	High sensitivity and specificity, no culture time needed	Expensive, complicated procedure, sensitive to experimental conditions	[10,32]
WGS	~2 d	Accurate, sensitive	Complex data analysis, expensive, time-consuming	[33]
MALDI-TOF	4 h	Rapid, inexpensive, simple procedure	Lack of specificity, database limitations, prolonged incubation time	[24,34,35]

**Table 2:** Single-stranded oligonucleotide probe and PCR forward and reverse primer sequences targeting the *bla*_NDM-1_ and *bla*_OXA-1_ CR genes, length, and melting temperature.

CR Gene	Probe and Primer Sequences (5′ to 3′)	Length (bp)	Tm (°C)
*bla*_NDM-1_			
	Probe 1: CAACACAGCCTGACTTTCGCCGCCAATGGCTGGGTCGAACCAGCAACCGC	50	74.4
	Probe 2: TGGCCCGCTCAAGGTATTTTACCCCGGCCCCGGCCACACCAGTGACAATA	50	74.5
	F-Primer: ATGGAATTGCCCAATATTAT [41]	20	55.6
	R-Primer: TCAGCGCAGCTTGTCGGCCA [41]	20	71.0
*bla*_OXA-1_			
	Probe: CGATGCATCCACAAACGCTGAAATTGCTCAATTCAATAAAGCAAAGTGTG	50	66.2
	F-Primer: ATATCTCTACTGTTGCATCTCC [42]	22	59.3
	R-Primer: AAACCCTTCAAACCATCC [42]	18	57.5

## Data Availability

The data presented in this study are available upon request from the corresponding author.
